# Moderate Antiproteinuric Effect of Add-On Aldosterone Blockade with Eplerenone in Non-Diabetic Chronic Kidney Disease. A Randomized Cross-Over Study

**DOI:** 10.1371/journal.pone.0026904

**Published:** 2011-11-04

**Authors:** Lene Boesby, Thomas Elung-Jensen, Tobias Wirenfeldt Klausen, Svend Strandgaard, Anne-Lise Kamper

**Affiliations:** 1 Department of Nephrology, Herlev Hospital, University of Copenhagen, Herlev, Denmark; 2 Department of Nephrology, Rigshospitalet, University of Copenhagen, Copenhagen, Denmark; 3 Department of Haematology, Herlev Hospital, University of Copenhagen, Herlev, Denmark; Universidade de Sao Paulo, Brazil

## Abstract

**Background:**

Reduction of proteinuria and blood pressure (BP) with blockers of the renin-angiotensin system (RAS) impairs the progression of chronic kidney disease (CKD). The aldosterone antagonist spironolactone has an antiproteinuric effect, but its use is limited by side effects. The present study evaluated the short-term antiproteinuric effect and safety of the selective aldosterone antagonist eplerenone in non-diabetic CKD.

**Study Design:**

Open randomized cross-over trial.

**Setting and Participants:**

Forty patients with non-diabetic CKD and urinary albumin excretion greater than 300 mg/24 hours.

**Intervention:**

Eight weeks of once-daily administration of add-on 25–50 mg eplerenone to stable standard antihypertensive treatment including RAS-blockade.

**Outcomes & Measurements:**

24 hour urinary albumin excretion, BP, p-potassium, and creatinine clearance.

**Results:**

The mean urinary albumin excretion was 22% [CI: 14,28], P<0.001, lower during treatment with eplerenone. Mean systolic BP was 4 mmHg [CI: 2,6], P = 0.002, diastolic BP was 2 mmHg [CI: 0,4], P = 0.02, creatinine clearance was 5% [CI: 2,8], P = 0.005, lower during eplerenone treatment. After correction for BP and creatinine clearance differences between the study periods, the mean urinary albumin excretion was 14% [CI: 4,24], P = 0.008 lower during treatment. Mean p-potassium was 0.1 mEq/L [CI: 0.1,0.2] higher during eplerenone treatment, P<0.001. Eplerenone was thus well tolerated and no patients were withdrawn due to hyperkalaemia.

**Limitations:**

Open label, no wash-out period and a moderate sample size.

**Conclusions:**

In non-diabetic CKD patients, the addition of eplerenone to standard antihypertensive treatment including RAS-blockade caused a moderate BP independent fall in albuminuria, a minor fall in creatinine clearance and a 0.1 mEq/L increase in p-potassium.

**Trial Registration:**

Clinicaltrials.gov NCT00430924

## Introduction

Reductions in blood pressure (BP) and urinary albumin excretion in chronic kidney disease (CKD) have been shown to reduce the risk of cardiovascular (CV) events and improve renal outcome. A reduction in proteinuria is considered a surrogate marker of reduction in CKD progression rate and residual proteinuria has influence on the course of progression to end stage renal disease [1]–[5].

Inhibition of the renin-angiotensin system (RAS) attenuates the progression of diabetic and non-diabetic CKD in patients with proteinuria and hypertension in excess of the BP lowering effect alone [6], [7]. There is increasing evidence that aldosterone has effects on the vascular wall leading to fibrosis, glomerular sclerosis and arterial stiffness, factors which in themselves lead to worsening of proteinuria and progression of CKD [8]–[10]. Inhibition of aldosterone by the non-selective antagonist spironolactone has been shown to reduce albuminuria in both diabetic and non-diabetic CKD [11]–[15]. The antiproteinuric effect of the selective aldosterone inhibitor, eplerenone, has previously been studied in type 2 diabetic patients with microalbuminuria [16] and in essential hypertension [17]. The aim of the present study was to evaluate the short-term effects of eplerenone in patients with non-diabetic CKD.

## Methods

### Ethics Statement

All patients were included after written informed consent. The study was approved by The Ethical Committee of Copenhagen County and the Danish Medicines Agency. The study was carried out according to the Helsinki Declaration.

The protocol for this trial and supporting CONSORT checklist are available as supporting information; see Checklist S1 and Protocol S1.

### Design

The study was carried out in a randomized, open-label, cross-over design comparing an 8-week control period with an 8-week period of once-daily administration of eplerenone. Randomization was done by the principal investigator drawing sealed opaque envelopes. Patients were either allocated to start in the intervention period followed by the control period or vice versa with no wash-out period in between.

### Study Participants

Inclusion criteria were: age >18 years, persistent 24 hour proteinuria, initially planned as >2000 mg, but after inclusion of the first patient changed to >500 mg or albuminuria >300 mg, BP>130/80 mmHg or ongoing stable antihypertensive treatment, including RAS-blockade. There was no demand for ongoing RAS-blocking therapy.

Exclusion criteria were: diabetic nephropathy, creatinine clearance <20 mL/min, plasma (p-) potassium >5.0 mEq/l, allergy to aldosterone antagonists, chronic liver insufficiency, ongoing treatment with CYP3A4-inhibitors, lithium or immunosuppressive agents including steroids, invalidating psychiatric disorders, other severe non-renal disease, woman of childbearing potential not using safe contraception, pregnancy or breast-feeding. Patients were recruited from and followed in the outpatient clinics of the two participating departments. All patients were seen by the principal investigator.

### Study protocol

Eplerenone treatment was initiated by a once daily oral dose of 25 mg administered as add-on treatment to ongoing therapy. The dose was doubled after one week to 50 mg once daily for seven weeks. Patients were seen at weeks 0, 1, 2, 4, 8, 9, 10, 12 and 16. The BP goal was <130/80 mmHg. In case of symptomatic hypotension, reductions were primarily made in non-RAS-blocking antihypertensive agents and in case of BP above target non-RAS-blocking agents were added. Main outcome variables were albuminuria based on single 24 hour urine samples collected for every visit, fractional excretion of albumin, BP, p-potassium, and creatinine clearance, which were measured at each visit.

### Safety and withdrawal criteria

Potassium supplements were withdrawn before initiation of eplerenone treatment. There were no dietary restrictions at trial entry. Withdrawal criteria were any CV event, serious non-renal disease, a persistent rise in p-creatinine above 30% of baseline value, persistent hyperkalaemia, defined as p-potassium >5.5 mEq/L at two successive visits, or pregnancy despite safe contraception.

### Clinical and biochemical methods

BP was measured using the mercury sphygmomanometer auscultatory method. Cuff width was selected according to arm circumference. BP measurements were done after 5–10 minutes of rest in the sitting position. Analysis of urinary albumin was done by immunoturbidimetry with antibodies from Dako A/S (Konelab PRIME 60), inter-series coefficient of variation (CV) 2% at Herlev Hospital and Tinaquant from Roche (Roche Modular), inter-series CV 10%, at Rigshospitalet.

P-creatinine was analyzed by the enzymatic method, MDRD-IDMS calibrated (Vitros 5.1), inter-series CV 2% at Herlev Hospital and by Roche Modular Enzymatic method, MDRD-IDMS calibrated, inter-series CV 2.1% at Rigshospitalet.

Fractional albumin excretion was calculated according to the following formula:
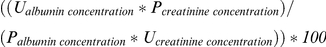
Compliance was monitored by tablet counts at the end of eplerenone treatment.

### Statistics

A reduction of albuminuria of 25% was considered as the minimal clinically relevant response to eplerenone treatment. Sample size was estimated to be a total of 40 patients, (power 0.8 and level of significance 0.05). Data are presented as mean and standard deviations (SD). Where data were not normally distributed, logarithmic transformations were made and these data are presented as well as geometric means with 95% confidence intervals [CI]. Changes in logarithmic transformed parameters were calculated as ratios and additionally shown in percent.

The treatment effects were calculated by the paired samples t-test, using the mean value of the variable over all four visits in each period – i.e. control and treatment. Analyses of values at individual visits were done as a secondary end-point. Before analyzing treatment effect, analyses of carry-over and time effect were made. To test for carry-over effect, we conducted a two-sample t-test between the two randomization groups using the mean value of each subject. For time effect a paired samples t-test was conducted comparing values for each patient at the two different time periods. Two-way ANOVA was used for testing the treatment effect on urinary albumin excretion when adjusting for creatinine clearance and systolic BP. One-way ANOVA was used for testing differences in response to treatment, when level of RAS-blockade or baseline creatinine clearance was taken into account. Evaluation of significance over multiple periods was done by repeated measure ANOVA. Comparisons of data from individual visits were corrected for multiple comparisons by the Bonferroni method. Since only baseline data were tested against data from each of the four following visits, correction was made by multiplying the P-values by four. When applying correction for multiple testing the corrected P-values are shown. All P-values were two-sided and P-values<0.05 were regarded as significant. All confidence intervals are two-sided 95% CI. The statistical analyses were done using the SPSS statistical software, version 17.0 and R statistical software version 2.12.1, 2011, R Foundation for Statistical Software, Vienna, Austria.

## Results

Forty-two patients were included in the study. One was included by mistake fulfilling the exclusion criterion of serious non-renal disease and was withdrawn after the first visit. Another patient was withdrawn due to non-compliance with the urinary collections. Thus, the statistical analysis comprises forty patients. Two patients were withdrawn before completion, one due to myocardial infarction (control period, study week 10) and one due to insomnia (treatment period, study week 14). Data from these two participants were averaged for the visits they had completed. Thirty-eight patients completed the full study protocol; one patient did not tolerate 50 mg eplerenone, as described below in the safety results. All patients demonstrated very good compliance, assessed by tablet counts. The trial was initiated in April 2007 and completed and ended as planned in August 2009.

### Baseline characteristics

Renal diagnoses were based on previous renal biopsies in chronic glomerulonephritis (n = 26), specified in table 1, and in vascular disease (n = 2). Autosomal dominant polycystic kidney disease (n = 1) was diagnosed by ultrasound scan. The remaining patients were categorized as having chronic nephropathy of unknown aetiology (n = 12). Patients with diabetic nephropathy were excluded.

**Table 1:** Baseline clinical data.

Baseline	Mean ± SD
	Geometric mean [95% CI]
**Gender ** ***(n)***	
Female/male	13/27
**Age, years**	45 (range, 21–71)
**Ethnicity ** ***(n)***	
Caucasian	37
African/Asian	3
**Renal Diagnosis ** ***(n)***	
Chronic glomerulonephritis#	26
Vascular disease	2
ADPKD§	1
CKD of unknown aetiology	11
**Antihypertensive medication ** ***(n)***	
ACE-inhibitor	23
Angiotensin Receptor Blocker	8
ACE-inhibitor and Angiotensin Receptor Blocker	7
Calcium Channel Blocker	10
Beta Blocker	6
Furosemide	14 Median 70 mg/day (range 20–250 mg)
Diuretic other	6
**BMI (kg/m^2^)**	27±4
**Systolic BP (mmHg)**	125±13
**Diastolic BP (mmHg)**	86±10

Note:

# IgA-nephropathy (n = 11), membranous nephropathy (n = 3), focal segmental glomerulosclerosis (FSGS) (n = 6), non-classified chronic glomerulonephritis (n = 6).

§ ADPKD = autosomal dominant polycystic kidney disease.

Creatinine clearance ranged from 24 to 195 mL/min at the time of inclusion and patients were classified as CKD stages 1 (n = 18), 2 (n = 10), 3 (n = 10) and 4 (n = 2). All patients but two were on RAS-blockade. One patient received vitamin D supplements. Baseline patient characteristics of all patients are presented in table 1 and 2. The median baseline number of antihypertensive drugs in the individual patient was two (range 0 to 5).

**Table 2:** Baseline laboratory data.

**P-albumin (g/dL)**	4.00±0.56
**P-cholesterol (mg/dL)**	216.6±34.8
**P-creatinine (mg/dL)**	1.53±0.63
**P-potassium (mEq/L)**	4.3±0.4
**Creatinine clearance (mL/min)** *	82 [71,96]
**Urine albumin excretion/24 hours (mg)** *	1624 [1310, 2015]

Note: Conversion factors for units: creatinine in mg/dL to µmol/L, ×88.4; cholesterol mg/dL to mmol/L, ×0.02586.

*Geometric mean and CI.

### Urinary Albumin Excretion

**Figure 1:**
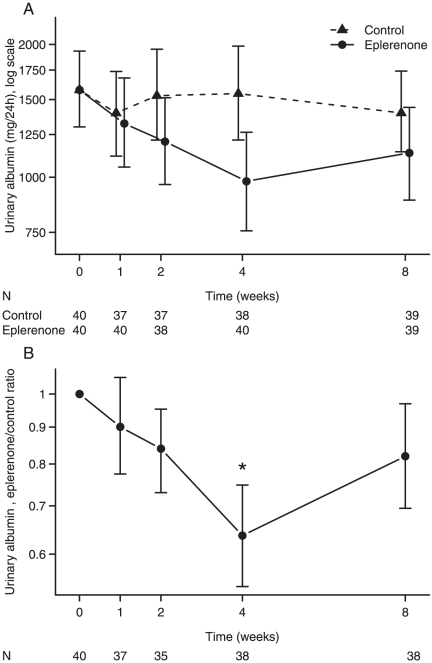
Urinary albumin excretion. Figure 1A illustrates albumin excretion during 8 weeks of add-on eplerenone as compared with control. Figure 1B illustrates the difference in urinary albumin excretion between treatment with eplerenone and control. Data are presented as mean values with 95% CI of the mean values. * P<0.05 for U-albumin excretion in the eplerenone period versus control period after Bonferroni correction. The difference in U-albumin excretion at week 4 versus week 8 in the eplerenone period was not significant. Note: N = number of urine samples.

Albuminuria was significantly lower during the add-on eplerenone period as compared with the control period with a 22% [CI: 14,28], P<0.001, lower excretion, as shown in table 3. The mean 24 hour excretion was 1481 mg [CI: 1192,1840] during the control period and 1163 mg [CI: 921, 1468] during add-on eplerenone. No significant carry-over, P = 0.3 or time effect, P = 0.3, was detected for the urinary albumin excretion. Reduction in albuminuria was observed in 31 of 40 patients. Mean changes in urinary excretion of albumin over time are shown in figure 1A, and the difference in albumin excretion during the two periods is illustrated in figure 1B. A significant decrease was seen after two weeks of add-on eplerenone, P = 0.003. Three patients had membranous nephropathy, a glomerular disease where resistance to aldosterone antagonists has been reported [18]. In two of these patients, a decreased urinary albumin excretion was observed during add-on eplerenone, while it was unchanged in the third patient.

**Table 3:** Results for Blood Pressure and Renal Parameters.

	Eplerenone	Control	Treatment effect	P-value
	Mean [95% CI]	Mean [95% CI]	Mean difference [95% CI]	
**Systolic BP (mmHg)**	121 [117,124]	124 [121,128]	−4 [−6,−2]	0.002
**Diastolic BP (mmHg)**	83 [80,86]	85 [82,88]	−2 [−4,0]	0.02
**P- albumin (g/dL)**	4.07 [3.89,4.24]	4.00 [3.83,4.17]	+0.07 [0.02,0.11]	0.004
**P- bicarbonate (mEq/L)**	23.9 [23.0,27.7]	24.3 [23.5,25.1]	−0.4 [−0.8,0.0]	0.05
**P- creatinine (mg/dL)**	1.56 [1.36,1.76]	1.51 [1.32,1.70]	+0.06 [0.02,0.09]	<0.001
**P- potassium (mEq/L)**	4.4 [4.3,4.5]	4.3 [4.2,4.4]	+0.1 [0.1,0.2]	<0.001
**P- urea nitrogen (mg/dL)**	29.97 [25.13,34.81]	26.85 [22.96,30.75]	+3.11 [1.31,4.92]	0.001
**Creatinine clearance**				
log10-values	1.91 [1.85,1.97]	1.93 [1.88,1.99]	−0.02 [−0.04,−0.01]	0.005
(mL/min)*	81 [71,93]	86 [75,97]	−5% [−2,−8]	0.005
**Urine albumin**				
log10-values	3.07 [2.96,3.17]	3.17 [3.07,3.27]	−0.11 [−0.15,−0.06]	<0.001
(mg/24hours)*	1163 [921,1468]	1481 [1192,1840]	−22% [−28,−14]	<0.001
**Fractional albumin excretion**				
log10-values	−1.60 [−1.73,−1.48]	−1.52 [−1.63,−1.40]	−0.09 [−0.13,−0.05]	<0.001
(%)*	0.025 [0.019,0.033]	0.030 [0.023,0.033]	−17% [−25,−11]	<0.001

Note: Conversion factors for units: creatinine in mg/dL to µmol/L, ×88.4; urea nitrogen mg/dL to mmol/L, ×0.357.

*Geometric mean and CI.

Percentages are obtained by subtracting log-values (treatment – control) and back-transforming by raising 10 to the difference. Values are mean values from visits at week 1, 2, 4 and 8 in the two time periods. P-values<0.05 are considered significant.

Fractional albumin excretion was significantly lower in the eplerenone period, see table 3, P<0.001.

When systolic BP reduction was taken into account, the urinary albumin excretion was 17% [CI: 8, 25] lower during eplerenone treatment, P<0.001. When corrected for the lower creatinine clearance during the treatment period, the reduction in albumin excretion was 19% [CI: 10,27], P = 0.0003. When both BP and creatinine clearance were included in the statistical model, the total difference in 24 hour urinary albumin excretion was 14% [CI: 4,24], P = 0.008.

As there was no wash-out period between study periods, additional analysis was done excluding the first measurements in each block (i.e. visit 2 and 6). Excluding these measurements the difference in urinary albumin excretion was 25% [CI: 17,32], P<0.001. Comparing only visits 2 and 6, there was no difference in albumin excretion between treatment and control periods, as well as there was no detection of any significant carry-over effect, P = 0.3.

To study the influence of baseline RAS-blockade on the antialbuminuric effect of eplerenone baseline RAS-blockade was separated into three levels for post hoc analysis: Low level blockade when the daily dose of ACEi/ARB was <¼ of the maximum dose for the individual drug (n = 11), moderate level blockade when the ACEi/ARB dose was between ¼ and ½ of the maximum dose (n = 18) and high level blockade when the ACEi/ARB dose was >½ the maximum dose (n = 2) or in case of combined ACEi and ARB (n = 7). There was a non-significant tendency for patients on moderate RAS-blockade to have a greater response on eplerenone than the other groups. Patients on combined ACEi and ARB had no significant reduction in albuminuria during eplerenone treatment.

### Blood pressure

Systolic and diastolic BP was significantly lower during add-on eplerenone treatment when compared to the control period (table 3). There was a significant reduction of systolic BP after two weeks of eplerenone treatment, P = 0.003. At later visits, there were no further significant changes in systolic BP. The diastolic BP was significantly reduced after four weeks of eplerenone treatment, P = 0.002, and there was a significant difference in diastolic BP between the treatment period and control period at the same time point, P = 0.004.

There were no significant differences between diastolic BP at the end of the two periods. There were no significant differences in BP reduction between groups of low, moderate or high baseline RAS-blockade.

There were no significant carry-over, P = 0.4 and P = 0.9, or time effects, P = 0.5 and P = 0.2 for systolic or diastolic BP.

### Safety results

As shown in table 3 creatinine clearance was 5% [2], [8], P = 0.005 lower in the eplerenone period compared to the control period. Mean creatinine clearance was 86 mL/min [CI: 75,97] during control and 81 mL/min in the eplerenone period [CI: 71,93]. The time course analyses showed that, the difference was only significant at week four of treatment versus week four of the control period, P = 0.03. There were modest increases in p-creatinine during add-on eplerenone, although no patients had an increase above 30%. Patients on baseline combined ACEi and ARB tolerated eplerenone as well as the other patients.

Four patients reported increased dizziness during the treatment period of the study compared to their usual state without any change in BP. None required reduction in dose of eplerenone due to dizziness alone.

No patients were withdrawn due to hyperkalaemia or other adverse events. The mean difference in p-potassium was 0.1 mEq/L, P<0.001 between the eplerenone and control periods, as shown in table 3.

Four percent of all p-potassium values measured were above 5.0 mEq/L and none were above 6.0 mEq/L. Only one patient had a single p-potassium value above 5.6 mEq/L at a planned visit. Consequently the patient was seen at an extra control, where p-potassium had increased to 5.8 mEq/L. Because of this, as well as symptomatic hypotension, nausea and general discomfort the dose of eplerenone was reduced to 25 mg daily. The patient completed the study without further adverse effects.

There were no significant carry-over effects, P = 0.3 and P = 0.1, or time effects, P = 0.7 and P = 0.7, for either creatinine clearance or p-potassium.

### Other parameters

Five patients had hypoalbuminemia, with a p-albumin <3.6 g/dL. There was a significantly higher concentration of albumin in plasma during treatment with eplerenone, P = 0.004. When the hypoalbuminemic patients were taken out of the statistical analysis, the P-value changed to 0.006. Twenty-nine patients had higher mean values of p-albumin during the treatment period, and 10 patients had lower values. Only one of the hypoalbuminemic patients had normalization of p- albumin in the treatment period and a 17% reduced urinary albumin excretion in the treatment period. Four patients were continuously hypoalbuminemic throughout the study. Two of these patients had a reduced excretion of albumin in urine and two had a higher excretion during treatment with eplerenone. Along with the analysis of changes in p-albumin it was also investigated whether there were any changes in p-cholesterol (total), p-LDL and p-HDL cholesterol and triglycerides. Neither of these changed significantly during the study. No patients were started or altered in lipid lowering treatment during the study period.

There were also no significant differences between study periods in the following parameters: weight, BMI, p-ionized calcium, p-uric acid, p-C-reactive-protein, blood haemoglobin, nor in the urinary excretion of creatinine, potassium and sodium.

## Discussion

The present study is the first to demonstrate an antiproteinuric effect of the selective aldosterone receptor antagonist, eplerenone, on overt albuminuria in non-diabetic CKD. Thus, in 40 CKD patients, add-on of eplerenone to stable antihypertensive treatment including RAS-blockade for eight weeks caused a 22% reduced excretion of urinary albumin compared to a control period. BP and creatinine clearance were slightly lower during the eplerenone period, but even after correction for that the urinary albumin excretion was significantly lower during treatment. The validity of the present observation rests on the randomized design of the study. The cross-over design was chosen in order to minimize the influence of heterogeneity between subjects and the lack of placebo control. The absence of a carry-over effect from eplerenone to control confirmed that no wash-out period after eplerenone was necessary.

Reduction in albuminuria during aldosterone blockade in the present study was less than that observed in the earlier studies. Despite this, it is relevant to consider addition of eplerenone in CKD patients on RAS-blockade, due to the fact that residual proteinuria influences the prognosis for proteinuric CKD [5].

The antiproteinuric effect of eplerenone has previously only been reported in patients with type 2 diabetes with microalbuminuria and in patients with essential hypertension [16], [17]. A study in type 2 diabetic patients, CKD stage 2 and microalbuminuria reported that add-on eplerenone to enalapril caused a marked reduction in urine-albumin-creatinine-ratio (UACR) of 41–48% [16]. In a study of essential hypertensive patients, it was found that 24 weeks of monotherapy with eplerenone caused a fall in UACR of 27–28% whereas the comparator amlodipine only caused a fall of 3–7% [17].

Studies with spironolactone have shown reduction in urinary protein excretion in the order of 30–58% in patients with diabetic and non-diabetic CKD stage 1–2 [11]–[15].

There may be two explanations for the antialbuminuric effect of aldosterone antagonism being smaller in our study than in the previous ones: First, in our study albuminuria was monitored in repetitive 24 hour urine samples collected for every visit and subsequently averaged, aiming at a high degree of accuracy of results. By contrast, some previous studies were based on a limited number of UACR measurements [16], [17]. Second, all our patients had overt albuminuria, while some of the patients in the earlier studies were selected to have microalbuminuria and many had normal renal function. Hence, our patients had a more severe burden of renal disease than the patients in the earlier studies, possibly restricting the effect of aldosterone antagonism on albuminuria.

The present study was not powered to detect differences between groups with different intensity of baseline RAS-blockade. Patients were on stable RAS-blockade in a dose at the discretion of their treating physician. In a post-hoc analysis we found that the reduction in proteinuria during add-on eplerenone tended to be most pronounced in patients treated at baseline with a moderate level dose of RAS-blockade. In a recently published study of diabetic patients with a UACR≥300 mg/g creatinine treatment was initially a supramaximal dose of ACEi. Spironolactone or an ARB was added for 48 weeks. Reduction in UACR was seen in both groups, but largest in the spironolactone group [19]. A study in patients with 24 hour proteinuria >1.5 g compared mono RAS-blockade (ACEi) to dual RAS-blockade (ACEi+ARB or ACEi+spironolactone) and triple RAS-blockade (ACEi+ARB+spironolactone). There was no further reduction when spironolactone was added to dual RAS-blockade [12]. In the present study there were a total of seven patients on dual blockade at baseline in whom no significant decrease of albumin excretion was seen. Of note this is at variance with other studies, that have shown a greater reduction in urinary protein excretion during triple RAS-blockade than during dual RAS-blockade [12], [20], [21].

Interestingly, an antiproteinuric effect of eplerenone was found in two out of the three patients with membranous nephropathy, a disease shown in a previous prospective study to be resistant to aldosterone inhibition [18].

As shown in figure 1A and B, there was a significant antialbuminuric effect after two weeks of add-on eplerenone.

A short-term study of aldosterone blockade with spironolactone in CKD has likewise shown an initial decrease in albuminuria after just two weeks of treatment [22].

**Figure 2:**
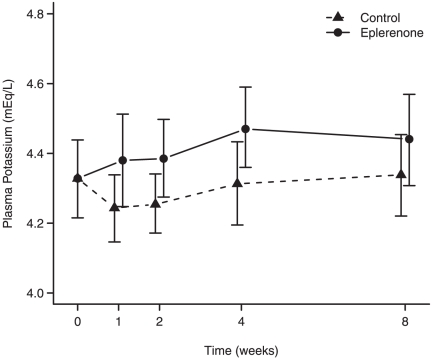
P-potassium during 8 weeks of add-on eplerenone as compared with control. Data are presented as mean values with 95% CI of the mean values.

P-potassium values rose during the treatment period. Figure 2 suggests that p-potassium may reach a steady level after four weeks of treatment with eplerenone.

The mechanisms involved in the antialbuminuric effect of aldosterone receptor blockade have not yet been fully elucidated. At present it is not known whether it is the classical or the non-classical (genomic or non-genomic) effects of aldosterone that influence progression of renal damage. An in vitro study has shown that eplerenone blocks the non-genomic pathways of aldosterone [23]. Other experimental studies have shown that damage to the podocytes can be induced by aldosterone and that this can be attenuated by eplerenone [10], [24], [25]. A possible indirect mechanism of reduced protein excretion is the effect of aldosterone on glomerular hemodynamics. Aldosterone has been suggested to augment glomerular filtration pressure, thereby increasing the filtration of macromolecules, a mechanism not blocked by spironolactone [23], [26], [27]. One experimental study has shown that non-genomic vasoconstriction mediated by aldosterone, was blocked by eplerenone [28].

The potential risks of blocking aldosterone receptors are mainly hypotension and hyperkalaemia. Previous studies initially reported no major clinical problems with hyperkalaemia [11]–[17], [29]–[31]. However studies reporting hyperkalaemia in the aftermath of study participation do exist [32].

Eplerenone in contrast to spironolactone does not cause gynecomastia or breast tenderness [33]. Gynaecomastia is in itself a common condition in the male CKD population and has been reported as a reason of discontinuation of treatment with spironolactone [11], [13], [29].

Hence, eplerenone may be a useful drug in proteinuric patients with CKD. The present study contributes to demonstrate eplerenone as an alternative to treatment with spironolactone and as a treatment alternative to patients who do not tolerate ACE-inhibition due to side effects.

### Study limitations

The group of patients studied were not homogeneous with respect to baseline albuminuria and renal diagnoses. There were no requirements to baseline therapy or run-in period. The study was not designed to detect a difference in response to eplerenone based on the baseline RAS-blocking treatment. It was an open label design and a relatively small sample size, which limits the extent to which results should be generalized. The statistical analyses were based on samples from all visits which were given equal weight in the analyses.

### Conclusion

Eplerenone in a dose of 25–50 mg daily is a safe and relevant add-on therapy in patients with non-diabetic CKD when aiming at a reduction in urinary albumin excretion. The potential risk of hyperkalaemia, especially in daily practice outside the setting of a controlled trial, should be kept in mind. Long-term studies need to be designed in order to assess the question of whether the effect of eplerenone on the reduction in urinary albumin excretion is persistent, and whether the progression rate of CKD is reduced.

## Supporting Information

Checklist S1 **An index of where in the article specific parts of the required information may be found.**
(DOC)

Protocol S1 **The study was carried out according to the protocol, which can be accessed here.**
(DOC)
