# Supplementary material for: Moderate Antiproteinuric Effect of Add-On Aldosterone Blockade with Eplerenone in Non-Diabetic Chronic Kidney Disease. A Randomized Cross-Over Study
Source: PLoS One. 2011 Nov 4;6(11):e26904. doi: 10.1371/journal.pone.0026904 (PMC3208556; doi:10.1371/journal.pone.0026904)
Supplement: Protocol S1 — The study was carried out according to the protocol, which can be accessed here. (DOC) [file pone.0026904.s002.doc]

# Aldosteronreceptorblokade med eplerenone ved kronisk nyresygdom.

# Tolerabilitet og effekt på proteinudskillelse i urin.

Læge Lene Boesby

Læge Thomas Elung-Jensen, Ph.D.

Overlæge Svend Strandgaard, Dr.med.

Overlæge Anne-Lise Kamper, Dr. med.

[Aldosteronreceptorblokade med eplerenone ved kronisk nyresygdom. 1](#__RefHeading___Toc174505394)

[Tolerabilitet og effekt på proteinudskillelse i urin. 1](#__RefHeading___Toc174505395)

[1. De klinisk ansvarlige, sponsor og investigators underskrift 4](#__RefHeading___Toc174505396)

[2. Lægmandsresume 5](#__RefHeading___Toc174505397)

[3. Resume 6](#__RefHeading___Toc174505398)

[4. Generelle oplysninger 7](#__RefHeading___Toc174505399)

[4.1 PROJEKTGRUPPE 7](#__RefHeading___Toc174505400)

[4.2 MONITORERING 8](#__RefHeading___Toc174505401)

[4.3 LOKALITETER OG TIDSRAMME 8](#__RefHeading___Toc174505402)

[5. Projektbeskrivelse 9](#__RefHeading___Toc174505403)

[5.1 FORMÅL 9](#__RefHeading___Toc174505404)

[5.2 EFFEKTPARAMETRE 9](#__RefHeading___Toc174505405)

[5.3 BAGGRUND 9](#__RefHeading___Toc174505406)

[5.4 METODE 11](#__RefHeading___Toc174505407)

[5.5 DESIGN 11](#__RefHeading___Toc174505408)

[5.6 PATIENTPOPULATION 13](#__RefHeading___Toc174505409)

[5.7 INKLUSIONSKRITERIER 13](#__RefHeading___Toc174505410)

[5.8 EKSKLUSIONSKRITERIER 14](#__RefHeading___Toc174505411)

[5.9 KRITERIER FOR UDTRÆDEN 14](#__RefHeading___Toc174505412)

[5.10 DEMOGRAFISKE VARIABLE 15](#__RefHeading___Toc174505413)

[5.11 PARAKINISKE VARIABLE 15](#__RefHeading___Toc174505414)

[5.12 PRAKTISK UDFØRELSE 16](#__RefHeading___Toc174505415)

[5.13 FORSØGSMEDICIN 16](#__RefHeading___Toc174505416)

[5.14 STATISTIK 17](#__RefHeading___Toc174505417)

[6. Kvalitetskontrol og kvalitetssikring 17](#__RefHeading___Toc174505418)

[7. Etiske overvejelser 18](#__RefHeading___Toc174505419)

[7.1 PROJEKTETS KONSEKVENS OG FORDELE 18](#__RefHeading___Toc174505420)

[7.2 PROJEKTETS ULEMPER OG RISICI 18](#__RefHeading___Toc174505421)

[7.3 SAMMENFATTENDE 19](#__RefHeading___Toc174505422)

[8. Økonomisk støtte 20](#__RefHeading___Toc174505423)

[9. Patientinformation og samtykkeerklæring 20](#__RefHeading___Toc174505424)

[10. Publikation og rapportering til myndighederne 21](#__RefHeading___Toc174505425)

[11. Forsikringsforhold 21](#__RefHeading___Toc174505426)

[12. Referencer 22](#__RefHeading___Toc174505427)

[Bilag Ia 24](#__RefHeading___Toc174505428)

[PATIENTINFORMATION – GENERELLE OPLYSNINGER 24](#__RefHeading___Toc174505429)

[Bilag Ib 25](#__RefHeading___Toc174505430)

[Patientinformation – om forsøget og dets forløb 25](#__RefHeading___Toc174505431)

[Bilag Ic 28](#__RefHeading___Toc174505432)

[Patientinformation – det praktiske omkring fremmøderne 28](#__RefHeading___Toc174505433)

[Bilag Id 29](#__RefHeading___Toc174505434)

[MØDEPLAN 29](#__RefHeading___Toc174505435)

[Bilag Ie 31](#__RefHeading___Toc174505436)

[SAMTYKKEERKLÆRING 1 31](#__RefHeading___Toc174505437)

[Bilag If 32](#__RefHeading___Toc174505438)

[SAMTYKKEERKLÆRING 2 32](#__RefHeading___Toc174505439)

[Bilag II 33](#__RefHeading___Toc174505440)

[STUDIEDESIGN 33](#__RefHeading___Toc174505441)

[Bilag III 34](#__RefHeading___Toc174505442)

[CASE REPORT FORM FOR DEMOGRAFISKE VARIABLE: 34](#__RefHeading___Toc174505443)

[Bilag III 35](#__RefHeading___Toc174505444)

[CASE REPORT FORM FOR BIOKEMI VED START OG KONTROLLER: 35](#__RefHeading___Toc174505445)

[Bilag IV 36](#__RefHeading___Toc174505446)

[Kildedataliste 36](#__RefHeading___Toc174505447)

[Bilag V 37](#__RefHeading___Toc174505448)

[Medicinregnskab per forsøgsperson 37](#__RefHeading___Toc174505449)

[BilagVI 38](#__RefHeading___Toc174505450)

[Algoritme for hyperkaliæmi og dosering af Inspra 38](#__RefHeading___Toc174505451)

[Bilag VII 39](#__RefHeading___Toc174505452)

[DINE RETTIGHEDER SOM FORSØGSPERSON – FRA VIDENSKABSETISK KOMITÉ 39](#__RefHeading___Toc174505453)

[Bilag VIII 40](#__RefHeading___Toc174505454)

[Curriculum Vitae 40](#__RefHeading___Toc174505455)

## 1. De klinisk ansvarlige, sponsor og investigators underskrift

Undertegnede klinisk ansvarlige er hermed ansvarlig for, at det beskrevne forsøg udføres i overensstemmelse med protokollen, gældende lov og GCP, samt at forsøget monitoreres af Københavns Universitetshospitals GCP-enhed. Forsøget kan blive del af ekstern audit.

………. ………………………………………………………………………….

Dato Thomas Elung-Jensen, klinisk ansvarlig

Protokolversion 6, 10.08.2007

………. ………………………………………………………………………….

Dato Svend Strandgaard, klinisk ansvarlig

Protokolversion 6, 10.08.2007

Undertegnede sponsor og investigator er hermed ansvarlig for, at det beskrevne forsøg udføres i overensstemmelse med protokollen, gældende lov og GCP, samt at forsøget monitoreres af Københavns Universitetshospitals GCP-enhed. Forsøget kan blive del af ekstern audit.

.

………. ………………………………………………………………………….

Dato Lene Boesby, investigator og sponsor

Protokolversion 6, 10.08.2007

## 2. Lægmandsresume

Hos patienter med kronisk nyresygdom vil der ofte ske en gradvis nedsættelse af nyrefunktionen over tid. Denne forværring sker i reglen for den enkelte patient med en konstant hastighed uafhængigt af hvilken nyresygdom, der er tale om. På et tidspunkt vil nyrefunktionen være så beskeden, at dialysebehandling eller nyretransplantation bliver nødvendig.

Denne fremadskriden i den kroniske nyresygdom kan delvis bremses ved medicinsk behandling. Det er vist at brug af ACE-hæmmere og Angiotensin receptor antagonister hæmmer progressionshastigheden. Mekanismen er ikke fuldt afklaret, men blodtryksregulation og nedsættelse af proteinurigraden spiller en vigtig rolle.

I dette forsøg ønskes at undersøge om tillæg af behandling med en såkaldt aldosteronreceptorhæmmer, har en yderligere nyrebeskyttende virkning. Aldosteron er et hormon, der produceres i binyrebarken.

Der er holdepunkter for, at aldosteron har en skadelig effekt på blodkarrene og dermed også på nyrefunktionen. Ved anvendelse af ACE-hæmmere og angiotensin receptor antagonister mindskes produktionen af aldosteron, men kun midlertidigt. Det kunne derfor være en fordel også at blokere aldosteron med et selvstændigt lægemiddel.

I undersøgelsen tillægges en aldosteronreceptorhæmmer, eplerenone, i den anbefalede dosering til patienternes sædvanlige medicin.

Undersøgelsen omfatter 40 patienter med kronisk nyresygdom. Forsøget strækker sig over 2 perioder på hver 8 uger. En lodtrækning afgør i hvilken periode deltageren skal behandles og i hvilken periode deltageren blot skal kontrolleres.

Aldosteronhæmning kan medføre ophobning af saltet kalium i blodet. Det er derfor nødvendigt at kontrollere dette omhyggeligt ved blodprøver. Således skal der tages ugentlige blodprøver de første to uger efter aldosteronhæmning er påbegyndt, og efterfølgende én gang hver fjerde uge. Ved alle fremmøder måles blodtryk. Der vil være 9 fremmøder i alt over 16 uger.

Deltageren vil få målt sin nyrefunktion ved analyse af opsamlet døgnurin ved starten af forsøget og ved efterfølgende kontroller.

Tidsforbruget og blodprøvetagning samt opsamling af døgnurin må opfattes som en mulig gene for patienten. Den hyppige kontrol i starten af forsøget er for at sikre opdagelse af svært forhøjet kaliumindhold i blodet. Behandling med eplerenone kan give andre bivirkninger, der anses som milde.

Projektet sponsoreres af de involverede afdelinger og medicinen udleveres af sygehusapoteket via afdelingen. Der er ingen tilknytning til medicinindustrien.

For den enkelte patient må forventes neutral effekt af deltagelse i undersøgelsen. For gruppen af patienter med kronisk nyresygdom må det forventes, at der opnås viden om forbedrede behandlingsmuligheder af kronisk nyresygdom, således at tidspunktet for påbegyndelse af dialysebehandling kan udskydes.

## 3. Resume

Titel: Aldosteronreceptorblokade med eplerenone ved kronisk nyresygdom. Tolerabilitet og effekt på proteinudskillelse i urin.

Formål: At vurdere effekten af aldosteronreceptorblokade på nyrefunktion ved brug af eplerenone, samt vurdere tolerabiliteten heraf hos patienter med kronisk nyresygdom. Som surrogatmål anvendes proteinudskillelse i urin.

Population: 35 patienter med kronisk nefropati. Alle patienter følges i Nefrologisk Ambulatorium på Amtssygehuset i Herlev eller på Rigshospitalet.

Udførelse: Metoden er en ublindet cross-over forsøg, hvor patienter randomiseres til enten at begynde med 8 ugers behandling med eplerenone i standard dosis efterfulgt af 8 ugers klinisk kontrol eller omvendt. Randomisering sker efter inklusion ved, at investigator trækker en lukket, ugennemsigtig kuvert fra en lukket pulje. I kuverten er det angivet, hvorvidt patienten skal starte i Inspragruppen eller kontrolgruppen. Patienter, der inkluderes på Rigshospitalet randomiseres af investigator på Herlev. Der er således kun et randomiseringssted.

For hver patient registreres følgende: køn, alder, højde, vægt, blodtryk, puls, creatininclearance målt ved døgnurin, proteinuri, nefrologisk diagnose, diagnosetidspunkt, medicinforbrug.

Ved forsøgets start tages følgende blodprøver: b-hæmoglobin, b-leukocytter, b-thrombocytter, p-bilirubin, p-ALAT, p-basisk fosfatase, p-cholesterol (total-, HDL- og LDL-), p-triglycerider, p-C-reaktivt protein, p-creatinin, p-carbamid, p-natrium, p-kalium, p-bikarbonat, p-fosfat, p-calcium-ion, blodsukker, p-albumin, p-urat samt døgnurinanalyse med henblik på baseline u-protein og creatininclearance. Hos kvinder tages ved 1. møde p-choriongonadotropin.

Ved øvrige kontroller tages alle ovennævnte blodprøver til analyse og der afleveres døgnurin, som undersøges for proteinudskillelse og creatininclearance.

Analyse: De indsamlede data gennemgår statistisk bearbejdning med Wilcoxon test for parrede data. En P-værdi < 0,05 anses for signifikant.

## 4. Generelle oplysninger

### 4.1 PROJEKTGRUPPE

Læge Lene Boesby er investigator og sponsor

Nefrologisk afdeling B

Amtssygehuset i Herlev

Herlev Ringvej 75

2730 Herlev

44 88 44 88 lokal 8-3634

E-Mail: lenboe03@heh.regionh.dk

Læge, Ph.D. Thomas Elung-Jensen er klinisk ansvarlig på Rigshospitalet

Nefrologisk Klinik P

Rigshospitalet

Blegdamsvej 9

2100 København Ø

35 45 35 45 lokal 5-0588

Overlæge, Dr. med. Svend Strandgaard er klinisk ansvarlig på Amtssygehuset i Herlev

Nefrologisk afdeling B

Amtssygehuset i Herlev

Herlev Ringvej 75

2730 Herlev

44 88 44 88 lokal 8-3705

Overlæge, Dr.med. Anne-Lise Kamper er medvejleder

### 4.2 MONITORERING

GCP-enheden

Københavns Universitetshospitals GCP-enhed

Amtssygehuset i Gentofte, opg.15, 2.

Niels Andersensvej 65

2900 Hellerup

Tlf: 39 77 72 78

### 4.3 LOKALITETER OG TIDSRAMME

Nefrologisk afdeling B

Amtssygehuset i Herlev

Herlev Ringvej 75

2730 Herlev

Nefrologisk Klinik P
Rigshospitalet

Blegdamsvej 9

2100 København Ø

Forsøget forventes initieret i foråret 2007.

Forventes anmeldt til Den Videnskabsetiske Komite for Købehavns Amt, Lægemiddelstyrelsen og Datatilsynet november 2006.

Afsluttes når alle patienter har gennemført i alt 16 ugers behandling og kontrol.

Den samlede forsøgsvarighed anslås til at være 2 år.

## 5. Projektbeskrivelse

### 5.1 FORMÅL

At vurdere betydningen af aldosteronhæmning for progression af kronisk nefropati belyst ved reduktion af proteinuri og ændring i creatininclearance over en 8 ugers periode.

Hypotesen er, at aldosteron har en fibrosefremmende egenskab, der medvirker til progression i kronisk nefropati. Ved hæmning af aldosteron kan opnås en progressionshæmmende effekt og dermed udsættelse af tidspunktet for dialysekrævende nyresvigt.

### 5.2 EFFEKTPARAMETRE

Den primære effektparameter er fraktionel albuminekskretion.

Endvidere beregnes creatininclearance og døgnurinalbuminudskillelse.

### 5.3 BAGGRUND

Kronisk nyreinsufficiens vil hos at stort antal patienter medføre terminal nyresygdom (end-stage renal disease (ESRD)). Progressionen fra let til terminal nyreinsufficiens sker i reglen med individuel, men konstant hastighed. Terminal sygdom behandles med nyretransplantation eller dialyse. Det er velkendt, at ikke alle patienter er egnet til transplantation eller opnår nyretilbud pga organmangel. Dialysebehandling er forbundet med øget mortalitet. Således var mortalitetsraten i 2004 på 22,3 % og 13,7 % for kroniske hæmodialysepatienter hhv. peritonealdialysepatienter[1]. Såfremt progressionen af nyreinsufficiens kan hæmmes, om end blot for en periode, vil der være meget vundet for den enkelte patient såvel som for samfundet.

Det antages, at progression af nyreinsufficiens skyldes flere faktorer, herunder vasoaktive hormoner, vækstfaktorer og cytokiner[2]. Det er vist, at blokering af renin-angiotensin-aldosteronsystemet ved hæmning af angiotensin-converting enzyme (ACE) eller ved blokering af angiotensin II-receptorer kan bremse udviklingen af terminal nyresygdom[3;4]. Denne behandling er nu implementeret som standard terapi til kronisk nyresyge.

Flere dyreeksperimentelle arbejder peger på, at aldosteron spiller en rolle i patogenesen ved progredierende kronisk nyresygdom[5]. Ud over aldosterons velkendte effekter som regulator af ekstracellulærvolumen (ECV) og kaliumhomeostasen, har aldosteron yderligere endokrine funktioner. Det er påvist, at aldosteron har indflydelse på vaskulær remodellering og kollagendannelse, dvs. øger karstørrelsen og stivheden af endothelcellerne og dermed måske øget proteinuri. [4]. Det antages, at proteinuri medfører glomerulosclerose, tubulointerstitiel inflammation og fibrose[4]. Således anerkendes reduktion i proteinuri som et godt effektmål i nyreprotektionsstudier.

Såfremt aldosteronfrigørelsen hæmmes eller aldosteronreceptorerne blokeres antages det, at aldosterons skadelige effekter kan reduceres.

Aldosteronreceptorer findes i andre væv end nyrerne og blokering af aldosteronreceptorer indgår nu som en del af den anbefalede behandling til post-myocardieinfarkt patienter, da aldosteronhæmning er vist at have en cardioprotektiv virkning[6;7].

Der er foretaget studier, hvor blokering af mineralocorticoidreceptorerne medfører langsommere progression i nyreinsufficiens end forventet uden behandling. I disse studier er anvendt spironolacton. Spironolacton er ikke selektivt for aldosteronreceptorer og har derfor bivirkninger i form af gynækomasti.

I forbindelse med start af eplerenone forventes et lille reversibelt fald i GFR og en stigning i plasmakalium[8], men ikke bivirkning i form af gynækomasti. Dette er set ved studier med spironolacton og også til dels i studier med eplerenone[9].

Forsøgslægemidlet eplerenones kinetik er undersøgt[10;11]. Eplerenone er en selektiv aldosteron-receptor antagonist og er deriveret fra spironolacton. Det er en kompetitiv hæmmer af aldosteron pga. binding til aldosteronreceptorer. Farmakokinetikken er undersøgt for doser mellem 10 og 1000 mg per os. Lægemidlet absorberes godt efter peroral administration, halveringstiden er mellem 2,2 og 9,4 timer[11]. Eplerenone metaboliseres via CYP3A4 til en inaktiv metabolit[10]. Der er udført doseringsstudier mhp. en- eller flergangs dosering og studier hos patienter med nedsat nyrefunktion. Hos sidstnævnte konkluderes det, at nyrefunktionen ikke har signifikant indflydelse på clearance af eplerenone, dvs. der ikke er belæg for dosisreduktion ved nedsat nyrefunktion[11]. Den anbefalede vedligeholdelsesdosis er 50 mg en gang dagligt[10]. Initialdosis er 25 mg en gang dagligt.

I forsøget øges patienternes eplerenonedosis efter 1 uge såfremt p-kalium og øvrige blodprøver tillader dette. I bilag VI ses algoritme for håndtering af eventuel forekomst af hyperkaliæmi. Dosis vil blive justeret i henhold til foreliggende anbefalinger[10].

Den største risiko ved anvendelse af eplerenone er hyperkaliæmi, hvorfor der er behov for tæt kontrol i initialfasen. Eventuelle kaliumtilskud vil blive seponeret før opstart af eplerenone. Det skønnes med disse forholdsregler, at det er forsvarligt at indgive eplerenone i anbefalet dosis til patienter med nedsat nyrefunktion.

Den nuværende anbefalede behandling af kronisk nyresygdom omfatter en hæmning af renin-angiotensin-systemet (RAS) med enten ACE-hæmmer eller angiotensin II-antagonist (RAS-blokade). Der er beskrevet en gavnlig effekt af at addere disse to lægemidler[12]. En mindre gruppe af patienter med kronisk nyresygdom får ikke RAS-blokade grundet bivirkninger. Såfremt der findes effekt af aldosteronhæmning, vil der således være et alternativt behandlingstilbud til denne patientgruppe.

### 5.4 METODE

Patienterne undersøges med blodprøver og døgnurinopsamlinger samt blodtryksmåling ved indgangen til studiet. Herefter monitoreres de med blodprøver og blodtryksmåling ved hvert besøg. Proteinurien bestemmes som base-line ved indgang til studiet samt ved hvert besøg.

### 5.5 DESIGN

Studiet er et randomiseret, ublindet cross-over studie forløbende over 16 uger, heraf 8 uger under behandling med eplerenone og 8 uger som kontrol.

I studiet skal indgå 40 patienter med kronisk nyreinsufficiens, som følges i afdelingernes ambulatorier.

Det tilstræbes, at den enkelte patients blodtryk skal være velreguleret (120-130/70-80 mmHg) og ligge konstant i hele undersøgelsesperioden. Alle patienter skal fortsætte deres vanlige antihypertensive behandling, herunder også uændret behandling med RAS-blokade, som langt de fleste patienter får. Såfremt den antihypertensive terapi skal intensiveres under forsøget tillægges betablokade, calciumantagonist eller methyldopa. Hvis blodtrykket for den enkelte patient bliver lavere end det fastsatte mål reduceres primært calcium-antagonister og betablokade, idet uændret RAS-blokade søges opretholdt, så vidt det er muligt.

Kalium seponeres dette forud for opstart af eplerenone.

Patienterne screenes med døgnurin. Randomiseres ved første møde. Mødeplanen er ens for de to grupper, da de enkelte patienter fungerer som deres egne kontroller.

Mødeplan for patienter randomiseret til gruppe1 – indleder med eplerenone.

Møde 1: Blodtryk måles med sphygmanometer tre gange, og gennemsnittet af de to sidste målinger noteres. Der tages blodprøver (b-hæmoglobin, b-leukocytter, b-thrombocytter, p-bilirubin, p-ALAT, p-basisk fosfatase, p-cholesterol (total-, HDL- og LDL-), p-triglycerider, p-C-reaktivt protein, p-creatinin, p-carbamid, p-albumin, p-kalium og p-natrium, p-fosfat, p-calcium-ion, p-bicarbonat, p-urat, blodsukker og for kvinder p-choriongonadotropin). Døgnurinanalyse med henblik på proteinuri og creatininclearance. Initiering af eplerenonedosering 25 mg dagligt.

Møde 2: 1 uge. Aflevering af døgnurin, blodtryksmåling og blodprøver. Dosisøgning til 50 mg afhængigt af blodprøvesvar.

Møde 3: 2 uger. Aflevering af døgnurin, blodtryksmåling og blodprøver.

Møde 4: 4 uger. Aflevering af døgnurin, blodtryksmåling og blodprøver.

Møde 5: 8 uger. Aflevering af døgnurin, blodtryksmåling og blodprøver. Seponering af eplerenone.

Møde 6: 9 uger. Aflevering af døgnurin, blodtryksmåling og blodprøver.

Møde 7: 10 uger. Aflevering af døgnurin, blodtryksmåling og blodprøver.

Møde 8: 12 uger. Aflevering af døgnurin, blodtryksmåling og blodprøver.

Møde 9: 16 uger. Aflevering af døgnurin, blodtryksmåling og blodprøver.

Mødeplan for patienter randomiseret til gruppe 2 – indleder med kontrol.

Møde 1: Her måles blodtryk med sphygmanometer tre gange, hvor gennemsnittet af de to sidste målinger noteres og blodprøver tages (b-hæmoglobin, b-leukocytter, b-thrombocytter, p-bilirubin, p-ALAT, p-basisk fosfatase, p-cholesterol (total-, HDL- og LDL-), p-triglycerider, p-C-reaktivt protein, p-creatinin, p-carbamid, p-albumin, p-kalium og p-natrium, p-fosfat, p-calcium-ion, p-bicarbonat, p-urat, blodsukker og for kvinder p-choriongonadotropin). Døgnurinanalyse med henblik på baseline u-protein og creatininclearance.

Møde 2: 1 uge. Aflevering af døgnurin, blodtryksmåling og blodprøver.

Møde 3: 2 uger. Aflevering af døgnurin, blodtryksmåling og blodprøver.

Møde 4: 4 uger. Aflevering af døgnurin, blodtryksmåling og blodprøver.

Møde 5: Aflevering af døgnurin, blodtryksmåling og blodprøver. Initiering af eplerenonedosering 25 mg dagligt. 8 uger.

Møde 6: Aflevering af døgnurin, blodtryksmåling og blodprøver. Dosisøgning til 50 mg afhængigt af blodprøvesvar. 9 uger.

Møde 7: 10 uger. Aflevering af døgnurin, blodtryksmåling og blodprøver.

Møde 8: 12 uger. Aflevering af døgnurin, blodtryksmåling og blodprøver.

Møde 9: 16 uger. Aflevering af døgnurin, blodtryksmåling og blodprøver. Seponering af eplerenone.

Døgnurinopsamling kan finde sted +/- dage i forhold til klinisk kontrol. Ved alle møder medbringes tomme pakninger og medicincompliance noteres.

Dosis af eplerenone øges efter algoritme (bilag VI).

### 5.6 PATIENTPOPULATION

Potentielle deltagere er patienter med kronisk nefropati, der behandles i nefrologisk afdeling, Amtssygehuset i Herlev eller Rigshospitalet. Kontakt til patienterne vil ske i forbindelse med indlæggelse eller ambulant kontrol. Patienter som opfylder inklusionskriterierne vil indgå i studiet. Der udbetales ikke honorar til deltagerne. Projektmedicin udleveres.

### 5.7 INKLUSIONSKRITERIER

Patienten kan indgå i studiet, såfremt følgende er opfyldt:

- alder over 18 år
- der er afgivet frivilligt skriftligt informeret samtykke
- der er proteinuri > 500 mg/døgn
- hypertension eller behandling med anti-hypertensiva

### 5.8 EKSKLUSIONSKRITERIER

Patienten kan ikke indgå i studiet hvis

- den nefrologiske grundsygdom er diabetisk nefropati
- GFR < 20 ml/min
- p-kalium ligger uden for referenceintervallet 3,5 til 5,0 mM
- allergi over for aldosteronreceptorantagonister
- kronisk leverinsufficiens
- er i behandling med kraftige CYP3A4-hæmmere (itraconazol, ketoconazol, ritonavir, nelfinavir, clarithromycin, telithromycin, nefazodon)
- er i behandling med lithium, ciclosporin, tacrolimus
- har arvelig galactoseintolerans, lactasemangel, glucose/galactosemalabsorption
- er gravid eller ammende
- er kvinde i den fertile alder og ikke anvender sikker anti-konception i form af p-piller, spiral eller depotgestagen
- demens eller anden psykisk tilstand, der umuliggør forståelse af undersøgelsesbetingelserne
- anden svær kronisk ikke-renal sygdom efter investigators skøn

### 5.9 KRITERIER FOR UDTRÆDEN

Forsøgsdeltagere vil blive ekskluderet fra studiet såfremt én af følgende tilstande skulle opstå:

- graviditet
- tilstødende alvorlig sygdom
- uacceptable bivirkninger
- intraktabel hyperkaliæmi
- kardiovaskulært event såsom AMI eller cerebral apopleksi
- stigning i p-creatinin på > 30 % i forhold til udgangsværdi [13]
- stigning i proteinudskillelse på >100 % i forhold til udgangsværdi
- ønske om udtræden fra deltagers side

Beslutning om udtræden af deltager kan ske ved investigator, klinisk vejleder eller medvejleder.

Ved udtræden fortsættes den vanlige ambulante kontrol.

### 5.10 DEMOGRAFISKE VARIABLE

For hver deltager registreres følgende: køn, alder, højde, vægt, blodtryk, puls, nefrologisk grundsygdom, diagnosetidspunkt, eventuelle andre diagnoser, medicinforbrug. For hver deltager registreres data i deres case report form (CRF, bilag III). Disse data betragtes alle som kildedata, jævnfør bilag IV. Deltagerne tildeles fortløbende numre ved indgangen til studiet.

### 5.11 PARAKINISKE VARIABLE

For hver deltager kvantiteres på døgnurin mængden af udskilt albumin, creatinin, natrium og kalium samt koncentrationerne heraf.

Blodprøver ved indgang til studiet omfatter følgende: (b-hæmoglobin, b-leukocytter, b-thrombocytter, p-bilirubin, p-ALAT, p-basisk fosfatase, p-cholesterol (total-, HDL- og LDL-), p-triglycerider, p-C-reaktivt protein, p-creatinin, p-carbamid, p-albumin, p-kalium og p-natrium, p-fosfat, p-calcium-ion, p-bicarbonat, p-albumin, p-urat, blodsukker og for kvinder p-choriongonadotropin).

Ved efterfølgende kontroller tages hver gang blodprøver som ovenfor fraset p-choriongonadotropin.

Døgnurin skal opsamles og analyseres ved hvert besøg. Disse data betragtes ligeledes som kildedata (bilag III)

Vedrørende blodprøver: Ved hver blodprøvetagning udtages 20-30 ml blod. Analysen sker på Klinisk Biokemisk afdeling, Amtssygehuset i Herlev eller Rigshospitalet, som rutineprøver.

Vedrørende døgnurinopsamling: Døgnurinopsamlinger afleveres til analyse på Klinisk Biokemisk afdeling som rutineprøver.

Vedr biobank: Datatilsynet har givet tilladelse til oprettelse af en biobank, hvori vil indgå blodprøver fra alle forsøgsdeltagere. Disse prøver kan i tilfælde af fejlslagne analyser anvendes. Prøverne opbevares ved minus 80 grader celsius i frysere med alarm på Amtssygehuset i Herlev.

Procedure vedrørende data ved studiets afslutning

Personhenførbare data opbevares i 10 år, hvorefter de vil blive destrueret. Data opbevares på Nefrologisk Afdeling B, Amtssygehuset i Herlev og Nefrologisk Klinik P, Rigshospitalet samt hos projektgruppen. Data vil kun være tilgængelige for projektgruppen.

### 5.12 PRAKTISK UDFØRELSE

Patienter, som er interesserede i at deltage i undersøgelsen informeres om studiet. Den skriftlige patientinformation udleveres og gennemgås og evt. samtykke underskrives (bilag Ia-f, VI)

Ved samtykke udleveres mødeplan for patienterne.

Demografiske data registreres.

Randomisering:

Når inklusionskriterier er opfyldt randomiseres patienter til umiddelbart at påbegynde behandling med eplerenone i 8 uger efterfulgt af 8 ugers kontrol eller 8 ugers kontrol efterfulgt af 8 ugers behandling med eplerenone. Lodtrækning foregår ved trækning af lukkede, ugennemsigtige kuverter af investigator. Randomiseringssvaret noteres i patientens case report form (CRF).

### 5.13 FORSØGSMEDICIN

Forsøgsmedicinen er eplerenone, som er en selektiv aldosteronreceptorantagonist. Præparatet er godkendt lægemiddel med produktresume godkendt af Lægemiddelstyrelsen. Produktresumeet vil være tilgængeligt på forsøgsstedet, når studiet initieres. Kommercielt tilgængeligt findes præparatet kun hos én udbyder.

Initial behandlingsdosis er anbefalet til 25 mg én gang dagligt med øgning til 50 mg dagligt efter 1 uge. Eplerenone tillægges patientens vanlige medicin.

Medicinen vil blive leveret i vanlige pakninger, som svarer til studiets varighed. Al udleveret medicin vil blive noteret i deltagerens CRF. Ved hvert besøg registreres den indtagne medicin i patientens medicinregnskab (bilag IV). Deltagere vil blive grundigt informeret om vigtigheden af indtagelse af projektmedicinen og at der vil blive ført regnskab med dette. Deltagernes hyppige fremmøder sikrer høj medicincompliance.

### 5.14 STATISTIK

Der foretages statistisk sammenligning af data i kontrolperioden med data i eplerenoneperioden ved anvendelse af Wilcoxon test for parrede data. Data angives som median og spændvidde. En P-værdi < 0.05 vurderes signifikant.

Styrkeberegning er foretaget efter nedenstående beregning:

Standard deviationen (SD) af gennemsnitlige forskel i albuminekskretionen er 1,118414.

På baggrund af disse data beregnes, at der minimum skal være

(n1 =n2 = 2 (t_2α_+ t_β,r_)^2^ SD^2^ / MIREDIF^2^ ),

dvs. n = 2 (20 * 0,05 + 0,80)^2^  * 1,118414^2^ / 0,25^2^ = 33

patienter for at finde en forskel i albuminekskretion på 25 % (α = 0,05, β = 0,80)[14].

For at tage højde for frafald i undersøgelsesperioden stiles der mod at inkludere 40 patienter.

Demografiske variable angives med median og spændvidde.

## 6. Kvalitetskontrol og kvalitetssikring

Almindelige procedurer for kvalitetskontrol og kvalitetssikring vil blive fulgt, jvf. ICH GCP Guidelines. Data vil blive monitoreret af Københavns Universitets GCP-enhed. Der vil i forbindelse med det informerede samtykke blive indhentet fuldmagt fra forsøgsdeltagerne til, at Den videnskabsetiske Komité, Lægemiddelstyrelsen og GCP-enheden kan få adgang til oplysninger om vedkommendes journal. En kopi af den foreslåede tekst vedlægges som bilag Ia og Ib.

## 7. Etiske overvejelser

### 7.1 PROJEKTETS KONSEKVENS OG FORDELE

Projektet vil ikke umiddelbart få konsekvens for behandlingen af den enkelte patient. Intentionen er, at projektet vil føre til større indsigt i, om blokering af aldosteronreceptorer hos patienter med kronisk nefropati, kan nedsætte progressionshastigheden af den kroniske nefropati.

### 7.2 PROJEKTETS ULEMPER OG RISICI

1. tidsforbrug
2. ubehag ved blodprøvetagning
3. ulempe ved døgnurinopsamling
4. bivirkninger i forbindelse med behandlingen, herunder risiko for hyperkaliæmi

Ad 1: Tidsforbruget er stort for den enkelte patient, men sikrer at interventionen er sikker, fordi monitorering af plasmakalium er hyppig. Derudover forventes de mange fremmøder at øge medicincompliance.

Ad 2: Ved blodprøvetagning udtages maksimalt 30 ml blod. Det udgør ingen risiko.

Ad 3: Ulempe at skulle opsamle døgnurin flere gange end i et normalt kontrolforløb.

Ad 4: Almindelige bivirkninger ved behandlingen kan være kvalme, diarre, svimmelhed, hypotension, nyrefunktionsnedsættelse og hyperkaliæmi. Nyrefunktionsnedsættelsen er reversibel. Hyperkaliæmi tages der højde for ved tæt monitorering.

Ifølge produktresumeet ses endvidere følgende ikke almindelige bivirkninger:

Pyelonephritis, eosinifili, dehydrering, hyperkolesterolæmi, hypertriglyceridæmi, hyponatriæmi, søvnløshed, hovedpine, ortostatisk hypotension, arteriel trombose i benet, atrieflimren, myokardieinfarkt, venstresidigt hjertesvigt, respiratoriske lidelser, lidelser i thorax og mediastinum, pharyngitis, flatulens og opkastning, kløe, øget svedtendens, rygsmerter, krampe i benene, asteni, utilpashed, stigning i carbamid (BUN), øget kreatinin.

Registrering og rapportering af bivirkninger følger den gældende lovgivning. Der refereres efterfølgende til de i *Bekendtgørelsen for kliniske forsøg med lægemidler på mennesker* definerede typer af bivirkninger. Uventede og alvorlige formodede bivirkninger (Suspected Unexpected Serious Adverse Reactions, SUSARs), der er enten dødelige eller livstruende, vil blive indberettet til Lægemiddelstyrelsen senest 7 dage efter, at sponsor-investigator har fået kendskab til dem, og senest 8 dage efter indberetningen vil sponsor-investigator meddele Lægemiddelstyrelsen alle relevante oplysninger om opfølgningen. Alle andre uventede og alvorlige formodede bivirkninger, vil ligeledes blive indberettet til Lægemiddelstyrelsen senest 15 dage efter, at sponsor-investigator har fået kendskab til disse. Alvorlige, formodede bivirkninger (Serious adverse reaction, SAR) vil blive indberettet én gang årligt til Lægemiddelstyrelsen. Alvorlige hændelser og alvorlige bivirkninger (Serious Adverse Events, SAE) vil blive indberettet én gang årligt til Den videnskabsetiske Komité. Hændelser og bivirkninger vil fremgå af den endelige rapport til Lægemiddelstyrelsen over forsøgets resultater.

Ved uacceptable bivirkninger vil forsøgsdeltageren ekskluderes fra forsøget. Patienten vil herefter overgå til sin vanlige medicin og blive fulgt i nefrologisk regi, så længe dette er påkrævet.

### 7.3 SAMMENFATTENDE

Projektets patientpopulation har risiko for at progrediere til terminal nyreinsufficiens. Blokering af renin-angiotensinsystemet er en veletableret nyreprotektiv behandling ved kronisk nyresygdom. Alle deltagere i projektet vil være i behandling med enten ACE-I eller AIIA forud for forsøgets start.

Patientpopulationen er nøje selekteret udfra in-og eksklusionskriterier for at minimere risici ved behandlingen. Der er meget hyppige kliniske samt parakliniske kontroller af behandlingen, således at eventuelle bivirkninger eller komplikationer til behandlingen hurtigt kan opdages. Registrering og rapportering af bivirkninger følger gældende lovgivning.

Sammenfattende bedømmes fordelene og ulemperne for den samlede forsøgsgruppe at balancere.

Projektet vil ikke blive sat i gang før godkendelse fra den regionale videnskabsetiske komité, Datatilsynet samt Lægemiddelstyrelsen er opnået.

Ingen forsøgsdeltager vil indgå i projektet, før vedkommende har afgivet frivilligt, skriftlig informeret samtykke.

Patienter som ikke ønsker at deltage i projektet, vil ikke blive afkrævet begrundelse, og patientens deltagelse eller ej vil ikke påvirke den videre kontrol eller behandling.

Undersøgelsen udføres i overensstemmelse med Helsinki-deklarationen.

## 8. Økonomisk støtte

Projektet udføres for afdelingernes egne midler. Således udleveres projektmedicinen fra henholdsvis Nefrologisk afdeling B, Amtssygehuset i Herlev samt Nefrologisk Klinik P, Rigshospitalet og udgifter hertil afholdes af disse afdelinger.

Projektet har ingen relation til medicinindustrien.

## 9. Patientinformation og samtykkeerklæring

Potentielle deltagere er patienter med kronisk nefropati, der behandles i nefrologisk afdeling, Københavns Amtssygehus i Herlev eller i nefrologisk afdeling, Rigshospitalet.

RETNINGSLINIER FOR AFGIVELSE AF DEN MUNDTLIGE INFORMATION OG INDHENTELSE AF SAMTYKKE.

**Inden informationssamtalen**

Ved en vanlig konsultation i vores ambulatorium vil patienten oplyses om projektet. Udviser patienten interesse, aftales der tid og sted til en informationssamtale. Før informationssamtalen vil patienten blive gjort opmærksom på, at der er tale om en forespørgsel om deltagelse i et videnskabeligt forsøg. Han gøres opmærksom på, at han ved informationssamtalen har muligheden for at medbringe en bisidder. Desuden oplyses, at patienten til hver en tid kan trække sig ud af projektet - uden forklaring -, uden at det på nogen måde vil kunne påvirke den nuværende eller fremtidige behandling af patienten. Han vil ved den indledende samtale gøres opmærksom på, at han har ret til betænkningstid efter informationssamtalen om projektet.

#### Informationssamtalen og betænkningstid

Informationssamtalen vil foregå i rolige uforstyrrede fysiske rammer på aftalt tid og sted. Informationen gives af en læge i projektgruppen. Patienten og eventuel bisidder informeres først mundtligt om projektet. Herefter udleveres den skriftlige information, som patienten får tilstrækkelig tid til at læse igennem. Efterfølgende besvares tvivlspørgsmål. Patienten informeres om muligheden for endnu en samtale med besvarelse af yderligere afklarende spørgsmål. Desuden oplyses om mulighed for betænkningstid før patienten underskriver samtykkeerklæringen. Hvis patienten indvilliger i at deltage, anmodes han om at aflevere samtykkeerklæringen inden en uge efter informationssamtalen. Patienten er til hver en tid velkommen til at kontakte projektgruppens læger.

**Inden projektstart**

Patienten får udleveret en underskrevet kopi af samtykkeerklæringen samt mødetidspunkt for undersøgelserne.

## 10. Publikation og rapportering til myndighederne

Protokollen meldes til hjemmesiden [www.clin.trials.gov](http://www.clin.trials.gov/) før initiering af rekruttering.

Resultaterne, såvel positive som negative, vil blive offentliggjort. Arbejdet vil blive indsendt til et internationalt anerkendt lægevidenskabeligt tidsskrift.

Artiklen skrives af Lene Boesby. Medforfattere er Thomas Elung-Jensen, Anne-Lise Kamper og Svend Strandgaard.

Efter projektets afslutning vil sponsor-investigator senest 90 dage efter underrette Lægemiddelstyrelsen samt Den videnskabsetiske Komité om, at projektet er afsluttet. Projektets resultat vil snarest muligt herefter blive indsendt til Lægemiddelstyrelsen jf. *Lov om lægemidler § 24a, stk. 2, nr.4.*

## 11. Forsikringsforhold

Ved patient dødsfald eller skade henvises til patientforsikringsordningen eller lov om erstatning for lægemiddelskader.

## 12. Referencer

1. Danish Society of Nephrology. Danish National Registry Report on Dialysis and Transplantation in Denmark 2004. 34. 2005.
Ref Type: Report

2. Epstein M: Aldosterone as a mediator of progressive renal disease: pathogenetic and clinical implications. Am J Kidney Dis 2001;37:677-688.

3. Kamper AL, Strandgaard S, Leyssac PP: Effect of enalapril on the progression of chronic renal failure. A randomized controlled trial. Am J Hypertens 1992;5:423-430.

4. Remuzzi G, Perico N, Macia M, Ruggenenti P: The role of renin-angiotensin-aldosterone system in the progression of chronic kidney disease. Kidney Int Suppl 2005;S57-S65.

5. Epstein M: Aldosterone and the hypertensive kidney: its emerging role as a mediator of progressive renal dysfunction: a paradigm shift. J Hypertens 2001;19:829-842.

6. Effectiveness of spironolactone added to an angiotensin-converting enzyme inhibitor and a loop diuretic for severe chronic congestive heart failure (the Randomized Aldactone Evaluation Study [RALES]). Am J Cardiol 1996;78:902-907.

7. Pitt B, Remme W, Zannad F, Neaton J, Martinez F, Roniker B, Bittman R, Hurley S, Kleiman J, Gatlin M: Eplerenone, a selective aldosterone blocker, in patients with left ventricular dysfunction after myocardial infarction. N Engl J Med 2003;348:1309-1321.

8. Schjoedt KJ, Rossing K, Juhl TR, Boomsma F, Rossing P, Tarnow L, Parving HH: Beneficial impact of spironolactone in diabetic nephropathy. Kidney Int 2005;68:2829-2836.

9. Epstein M: Aldosterone receptor blockade and the role of eplerenone: evolving perspectives. Nephrol Dial Transplant 2003;18:1984-1992.

10. Lægemiddelstyrelsen. Produktresumé for Inspra, filmovertrukne tabletter. 1-11. 15-12-2005.
Ref Type: Report

11. Ravis WR, Reid S, Sica DA, Tolbert DS: Pharmacokinetics of eplerenone after single and multiple dosing in subjects with and without renal impairment. J Clin Pharmacol 2005;45:810-821.

12. Kanno T, Nakamura T, Suzuki H: Add-on Angiotensin Receptor Blocker in Patients Who Have Proteinuric Chronic Kidney Disease and Are Treated With Angiotensin-Converting Enzyme Inhibitors. Clin J Am Soc Nephrol 2006;1:730-737.

13. Hou FF, Zhang X, Zhang GH, Xie D, Chen PY, Zhang WR, Jiang JP, Liang M, Wang GB, Liu ZR, Geng RW: Efficacy and safety of benazepril for advanced chronic renal insufficiency. N Engl J Med 2006;354:131-140.

14. Andersen B, Havsteen B, Riis P, Almind G, Bock E, Hørder M: Biostatistik; in Andersen D, Havsteen B, Riis P, Almind G, Bock E, Hørder M (eds): Sundhedsvidenskabelig Forskning- en introduktion. Copenhagen, FADL, 1999, pp 207-302.

## Bilag Ia

Aldosteronreceptorblokade med eplerenone ved kronisk nyresygdom.

Tolerabilitet og effekt på proteinudskillelse i urinen.

(INSPRA-STUDIET)

### PATIENTINFORMATION – GENERELLE OPLYSNINGER

Vi henvender os til dig for at spørge, om du vil deltage i et videnskabeligt forsøg.

På de næste sider vil vi beskrive, hvad forsøget går ud på.

Det er **helt frivilligt,** om du ønsker at deltage. Selv om du har besluttet, at du gerne vil deltage, kan du på ethvert tidspunkt trække dit tilsagn tilbage, **uden** at du skal komme med begrundelser og **uden** at det vil få konsekvenser for din videre behandling i nefrologisk afdeling. Dette gælder også, selv om du har skrevet under på, at du vil deltage.

Hvis du ikke vil være med, respekterer vi naturligvis dette og du vil under alle omstændigheder fortsætte med at modtage den almindeligt anbefalede behandling.

Hvis du vil deltage i forsøget, beder vi dig skrive under på den vedlagte samtykkeerklæring. Vi anmoder om at få dette papir med din underskrift. Du får også selv et eksemplar af patientformationen.

Du får også folderen ”Dine rettigheder som forsøgsperson”, som vi anbefaler dig at læse, inden du giver dit eventuelle samtykke.

Forsøget er godkendt af Den Videnskabsetiske Komité for Københavns Amt.

## Bilag Ib

Inspra-studiet

### Patientinformation – om forsøget og dets forløb

Baggrund

Hos patienter med kronisk nyresygdom, vil nyrernes funktion i reglen aftage med tiden. Denne udvikling kan sjældent forhindres, men ofte bremses. I dag gøres dette med forskellig slags medicin. Det er vores ønske at undersøge, om tillæg af endnu et lægemiddel kan have en gavnlig virkning på kronisk nyresygdom Det er kendt, at mængden af æggehvidestoffer (protein) i urinen kan anvendes til vurdering af nyresygdom. Det er derfor måling af dette, vi vil anvende til at vurdere, om der er effekt af den nye behandling.

Formål

Eplerenone er en type godkendt medicin, som i dag bruges til behandling af hjertesvigt og som vanddrivende medicin. Formålet med vores forsøg er at undersøge, om eplerenone nedsætter tabet af æggehvidestof (protein) i urinen ved kronisk nyresygdom.

Forsøgsdeltagere

I forsøget deltager i alt 40 patienter for, at det kan afgøres, om lægemidlet har den forventede effekt eller ej. Alle deltagere skal have kronisk nyresygdom og tab af æggehvide i urinen. Alle deltagere bliver så vidt muligt set af den samme læge ved hvert besøg. Ved uacceptable bivirkninger, tilstødende anden alvorlig sygdom eller ved patientens ønske om at stoppe med forsøgsmedicinen udgår deltageren af forsøget.

Forsøgsforløb

For at kunne vurdere virkningen af forsøgsmedicinen, er det vigtigt, at der for hver patient er en periode, hvor man får eplerenone og en periode, hvor man udelukkende får sin vanlige medicin. Det afgøres ved lodtrækning, om man skal starte med 8 uger med eplerenone eller 8 uger uden eplerenone. Studiet varer derfor 16 uger i alt, hvor vi beder dig møde frem i alt 9 gange.

Der er ingen sikker direkte fordel for dig ved at deltage i studiet.

Ulemperne består i, at du skal bruge tid på at samle døgnurin og komme til ambulant kontrol hyppigere end du plejer samt, at der skal tages blodprøver.

Eplerenone er et gennemprøvet lægemiddel. Risikoen ved at deltage i studiet kan være, at du vil ophobe saltet kalium i blodet, hvorfor det er nødvendigt i begyndelsen at tage blodprøver hver uge.

***Bilag Ib***

Deltagelse i forsøget indebærer således:

- 9 kontrolbesøg inkl. blodtryksmåling og blodprøvetagning
- opsamling af døgnurin i alt 9 gange
- indtagelse af den udleverede medicin

Fordele, ulemper, bivirkninger og risici

Ulemperne ved at deltage i forsøget er tidsforbruget samt blodprøvetagningen og urinopsamlingen. Der kan være bivirkninger ved indtagelse af medicinen, men disse er oftest milde. Der kan eventuelt ske et lille fald i nyrefunktionen, når man begynder behandlingen, men dette forsvinder, når medicinen tages væk. Den væsentligste risiko er ophobning af kalium i blodet. For at sikre dig mod dette tages blodprøver ugentligt de første 4 uger af behandlingen. Endvidere er der følgende almindelige bivirkninger: kvalme, diarre, svimmelhed, lavt blodtryk, midlertidig nyrefunktionsnedsættelse og følgende ikke almindelige bivirkninger: nyrebækkenbetændelse, allergi, væskemangel, forhøjet kolesterol og fedtsyrer i blodet, lavt saltindhold i blodet, søvnløshed, hovedpine, blodtryksfald ved overgang til stående stilling, blodprop i benet, hjerteflimmer, blodprop i hjertet, hjertesvigt, lidelser i lungerne, brystkassen og brysthulen, halsbetændelse, oppustethed, opkastning, kløe, øget svedtendens, rygsmerter, krampe i benene, træthed, utilpashed, stigning i nyretal (carbamid og kreatinin).

Såfremt der skulle opstå uacceptable bivirkninger eller anden alvorlig tilstødende sygdom forbeholder forsøgsgruppen sig ret til at tage dig ud af forsøget.

Risikoen ved at deltage skønnes at være minimal, når man tager den tætte kontrol i betragtning.

Der er mulighed for, at forsøgsbehandlingen vil være til fordel for dit helbred. Som udgangspunkt kan man dog ikke forvente, at der er nogen direkte fordel for dig ved at deltage, ud over den viden forsøget vil bidrage med for nyrepatienter i almindelighed.

Særlige forholdsregler

VIGTIGT! Prednisolon, Prednison eller medicin af typen Non-Steroide Antiinflammatoriske farmaka (NSAID, der populært kaldes gigtmedicin) må **ikke** indtages i forsøgsperioden. Dette gælder både tabletter og diverse geler, salver e. lign. indeholdende disse indholdsstoffer. NSAID er præparater, der ofte er håndkøbsmedicin så som Brufen, Diclon, Ibumetin, Ibuprofen, Ipren, Voltaren m.fl.

**Disse præparater kan direkte skade nyrefunktionen i forbindelse med projektmedicinen.**

Mod hovedpine o. lign. kan istedet tages tablet Paracetamol / Panodil ½-1 gram op til 3-4 gange i døgnet.

Endvidere vil vi bede om, at du oplyser det til os, hvis du får ordineret andre receptpligtige lægemidler i forsøgsperioden.

***Bilag Ib***

Forventninger til forsøgsdeltager

Vi forventer af forsøgsdeltageren, at du

- indtager forsøgsmedicinen som foreskrevet
- møder til de aftalte kontroller på de aftalte tidspunkter
- oplyser om eventuelle bivirkninger

Økonomi

Udgifter til dette forsøg afholdes af Nefrologisk afdeling B, Herlev Hospital og Nefrologisk Klinik P, Rigshospitalet i fællesskab. Forskerne har ingen økonomiske interesser i forsøget. Der er ingen tilknytning til lægemiddelindustrien.

Hvis du vil vide mere, kontakt da venligst

Lene Boesby Thomas Elung-Jensen

Nefrologisk afdeling B Nefrologisk klinik P
Herlev Hospital Rigshospitalet

Tlf. 44 88 44 88 lokal 8-3634 Tlf. 35 45 35 45 lokal 5-0588

e-mail: [lenboe03@heh.regionh.dk](mailto:lenboe03@heh.regionh.dk)

## Bilag Ic

Inspra-studiet

### Patientinformation – det praktiske omkring fremmøderne

Første besøg:

Første gang du kommer i ambulatoriet efter, du har sagt ja til at deltage i forsøget, skal du aflevere en døgnurin. Du skal fortsætte med at tage din vanlige medicin som du plejer. Du taler med læge og får målt blodtryk og puls samt får taget blodprøver. Der vil blive trukket lod, om du skal begynde forsøgsperioden med at få forsøgsmedicinen eller om du skal begynde forsøgsperioden uden.

Besøget foregår i Nefrologisk ambulatorium.

Første besøg tager højst 1 time.

Øvrige besøg:

De efterfølgende gange vil du hver gang skulle medbringe døgnurin samt medicin-emballage. Ved hvert besøg skal du hvile 10 minutter før du får målt dit blodtryk. Hver gang skal du have taget blodprøver. Vi registrerer, at du har taget medicinen som planlagt. Ud fra de blodprøver du får taget, justerer vi medicinen i den 8 ugers periode du skal tage den.

Besøgene forventes at tage højst 30 minutter.

## Bilag Id

### **MØDEPLAN**

| **Besøgsnumer** | **Dato** | **Medicin**  **Inspra** | **Undersøgelser** | **Husk** |
| --- | --- | --- | --- | --- |
| 0 |  | mg | Klinisk undersøgelse, blodtryk, puls,  aflevere døgn-urinopsamling, **OBS 2 glas**,  blodprøver, lodtrækning. | - at få udleveret medicin  - at få dunk til døgnurinopsamling. |
| 1 |  | mg | Blodtryk, puls, aflevere døgnurinopsamling **OBS 2 glas**. | - at få udleveret medicin  - at få dunk til døgnurinopsamling  - at aflevere tom medicinemballage |
| 2 |  | mg | Blodtryk, puls, aflevere døgnurinopsamling **OBS 2 glas**. | - at få udleveret medicin  - at få dunk til døgnurinopsamling  - at aflevere tom medicinemballage |
| 3 |  | mg | Blodtryk, puls, aflevere døgnurinopsamling **OBS 2 glas**. | - at få udleveret medicin  - at få dunk til døgnurinopsamling  - at aflevere tom medicinemballage |
| 4 |  | mg | Blodtryk, puls, aflevere døgnurinopsamling **OBS 2 glas**. | - at få udleveret medicin  - at få dunk til døgnurinopsamling  - at aflevere tom medicinemballage |
| 5 |  | mg | Blodtryk, puls, aflevere døgnurinopsamling **OBS 2 glas**. | - at få udleveret medicin  - at få dunk til døgnurinopsamling  - at aflevere tom medicinemballage |
| 6 |  | mg | Blodtryk, puls, aflevere døgnurinopsamling **OBS 2 glas**. | - at få udleveret medicin  - at få dunk til døgnurinopsamling  - at aflevere tom medicinemballage |
| 7 |  | mg | Blodtryk, puls, aflevere døgnurinopsamling **OBS 2 glas**. | - at få udleveret medicin  - at få dunk til døgnurinopsamling  - at aflevere tom medicinemballage |
| 8 |  | mg | Blodtryk, puls, aflevere døgnurinopsamling **OBS 2 glas**. | - at få udleveret medicin  - at få dunk til døgnurinopsamling  - at aflevere tom medicinemballage |
| 9 |  | mg | Blodtryk, puls, aflevere døgnurinopsamling **OBS 2 glas**. | - at få udleveret medicin  - at aflevere tom medicinemballage |

## Bilag Ie

### SAMTYKKEERKLÆRING 1

Jeg bekræfter hermed, at jeg efter at have modtaget ovenstående information såvel mundtligt som skriftligt, indvilliger i at deltage i det beskrevne forsøg.

Jeg er informeret om, at det er frivilligt at deltage, og at jeg når som helst kan trække mit tilsagn om deltagelse tilbage, uden at det på nogen måde vil kunne påvirke den nuværende eller fremtidige lægelige behandling af mig.

Jeg giver hermed fuldmagt til, at min journal kan blive set af personer fra Lægemiddelstyrelsen, Den videnskabsetiske Komité samt GCP-enheden (GCP= Good Clinical Practice) ved Københavns Universitetshospital og andre relevante udenlandske myndigheder i 10 år efter forsøgets afslutning, i forbindelse med inspektion, audit og monitorering af forsøget.

Jeg giver tilladelse til, at min egen læge kan informeres om min forsøgsdeltagelse.

JA_____ NEJ_____

Deltagernavn………………………………………… Deltagernummer…………….

(blokbogstaver)

Dato………………. Underskrift……………………………………………………

Ovenstående fuldmagter gælder kun oplysninger, der har relation til forsøget og kan til enhver tid tilbagekaldes. Alle oplysninger bliver behandlet fortroligt.

Undertegnede bekræfter herved, at ovenstående information er givet såvel mundtligt som skriftligt, og at patienten indvilliger i at deltage i det beskrevne forsøg.

Dato…………….. Underskrift………………………………………………….

Informerende læge

## Bilag If

### SAMTYKKEERKLÆRING 2

Jeg bekræfter, at jeg har deltaget i ovenstående projekt.

I forbindelse med projektet er der taget blodprøver og urinprøver. En mindre del af prøverne er blevet frosset ned til brug for senere undersøgelser.

Jeg giver hermed tilladelse til, at det gemte materiale bruges:

_____ til forskning indenfor nyresygdomme generelt

_____ til forskning indenfor sygdomme generelt

(sæt kryds, gerne flere)

Jeg er informeret om, at jeg godt kan deltage i INSPRA-studiet uden at give tilsagn om nedfrysning af blod/urinprøver til senere brug. Jeg er ligeledes informeret om, at jeg når som helst kan trække dette tilsagn tilbage, uden at det på nogen måde vil kunne påvirke den nuværende eller fremtidige lægelige behandling af mig.

Jeg vil få udleveret en underskrevet kopi af denne samtykkeerklæring

…………………………………………………………..

Deltager navn (blokbogstaver)

................ …………………………………………………………..

Dato Deltager underskrift

Undertegnede bekræfter hermed, at ovenstående information er givet såvel mundtligt som skriftligt.

............... …………………………………………………………..

Dato Informerende læge

## Bilag II

### STUDIEDESIGN

## Bilag III

### CASE REPORT FORM FOR DEMOGRAFISKE VARIABLE:

Dato___________ Patientnummer____________

Navn__________________________________________ CPR-nummer_____________

Periode_____________ Forsøgsmedicin__________________________________

Køn_________ Alder________ Vægt_________ Højde_________

BT__________ Puls_________ Proteinuri_____ Clearance______

Nefrologisk diagnose______________________________________________________________

Andre diagnoser__________________________________________________________________

_______________________________________________________________________________

_______________________________________________________________________________

Medicin________________________________________________________________________

_______________________________________________________________________________

_______________________________________________________________________________

_______________________________________________________________________________

_______________________________________________________________________________

_______________________________________________________________________________

_______________________________________________________________________________

Kommentarer____________________________________________________________________

_______________________________________________________________________________

## **Bilag III**

### CASE REPORT FORM FOR BIOKEMI VED START OG KONTROLLER:

Besøgsnummer_________________

Dato__________ Patientnummer_________________

Navn_____________________________________ CPR-nummer__________________

Periode_________ Forsøgsmedicin______________________________________

b-hæmoglobin_____ b-leukocytter ______ b-thrombocytter_______

CRP_____________ p-natrium________ p-kalium_____________

p-creatinin__________ p-carbamid_______ p-albumin____________

p-calcium ion_______ p-urat___________ p-bicarbonat__________

p-fosfat____________

p-bilirubin_________ p-bas. fosfatase_____ p-ALAT____________

p-cholesterol_______ p-HDL____________ p-LDL_____________

p-triglycerid_______ p-glukose__________ p-HCG_____________

U-albumin, mængde___________ U-albumin, konc.________

U-kalium, mængde____________ U-kalium, konc._________

U-natrium, mængde____________ U-natrium, konc._________

U-creatinin________ U-creatinin, konc______ creatininclearance_______

Diurese__________ml/døgn

## Bilag IV

### Kildedataliste

Protokol: Aldosteronreceptorblokade med eplerenone ved kronisk nyresygdom. Tolerabilitet og effekt på proteinudskillelse i urin

Center: Medicinsk Nefrologisk afdeling B, Amtssygehuset i Herlev og Nefrologisk Klinik P, Rigshospitalet

Investigator: Lene Boesby

| *Data* | *Kilde* |
| --- | --- |
| Samtykke | Samtykkeerklæring |
| Demografi | Journal |
| Anamnese | Journal |
| Patient nummer | Fax fra sponsor |
| Besøgsdato | Journal |
| EKG | EKG-udskrift |
| Blodtryk | CRF |
| BMI | Udregnes ud fra journaldata |
| Blodprøver | Laboratorierapport |
| Compliance | Medicin regnskab |
| Primæreffektparameter | Laboratorierapport |
| Bivirkninger/hændelser | Journal |
| Anden medicin | Journal |

Investigator: Lene Boesby

Signatur: _____________________________ Dato:_____________

## Bilag V

### Medicinregnskab per forsøgsperson

Investigator: ______________________

Center:___________________________

| Medicinid  *Navn, batchnr, kode* | Mængde udleveret  *Antal pakninger/tabletter* | Udleveret til  *Forsøgsperson nr/id* | Udleveret af  *Dato og initialer* | Mængde returneret til destruktion  *Antal pakninger/tabletter* | Retur til  *Dato og initialer* |
| --- | --- | --- | --- | --- | --- |
|  |  |  |  |  |  |
|  |  |  |  |  |  |
|  |  |  |  |  |  |
|  |  |  |  |  |  |
|  |  |  |  |  |  |
|  |  |  |  |  |  |
|  |  |  |  |  |  |
|  |  |  |  |  |  |
|  |  |  |  |  |  |

**Til vitterlighed for dette dokument:**

*(udfyldes ved forsøgsafslutning)*

Investigator:

| _________ | ___________________________________ | ______________________________ |
| --- | --- | --- |
| Dato | Navn | Signatur |

## BilagVI

### Algoritme for hyperkaliæmi og dosering af Inspra

Hyperkaliæmi vil i øvrigt blive behandlet efter vanlige retningslinjer.

## Bilag VII

### DINE RETTIGHEDER SOM FORSØGSPERSON – FRA VIDENSKABSETISK KOMITÉ

## Bilag VIII

### Curriculum Vitae

# Personlige oplysninger

# Navn Lene Boesby

# Adresse Traneholmen 16 B

# 3460 Birkerød

# Telefon 33 79 32 78

# Mobil 24 91 11 19

# E-mail [lene@boesby.dk](mailto:lene@boesby.dk)

# Teoretisk uddannelse

# Lægevidenskabelig Embedseksamen januar 2004

# Københavns Universitet

# Studentereksamen, matematisk-kemisk linie 1990

# Ordrup Gymnasium

# Ansættelser

# Uklassificeret stilling Nefrologisk Afdeling B, KAS Herlev 1.9.06-

# Introduktionsstilling Medicinsk gastroenterologisk Klinik CA, RH 1.3.06 -31.8.06

# Hepatologisk Klinik A

# Nefrologisk Klinik P, RH 1.9.05- 28.2.06

# Medicinsk Endokrinologisk Klinik PE

# Turnus Niels Jerne Borrild, Ringtoften, Skovlunde 1.3.05-31.8.05

# Medicinsk Endokrinologisk afdeling J, KAS Herlev 1.9.04-28.2.05

# Kirurgisk Urologisk afdeling H, KAS Herlev 1.3.04-31.8.04

# Videnskabelige foredrag L Boesby, B Kromann-Andersen, B Edwin

# Erfaringer med laparoskopiske donornefrektomier

# Dansk Urologisk Selskab, Kolding, november 2004

# Publikationer L Boesby, B Kromann-Andersen, B Edwin, J Melchior Hansen

# *Erfaringer med laparoskopisk donornefrektomi på Amtssygehuset i Herlev*

# Antaget til publikation i Ugeskrift for læger

# (Originalarbejde)

#

# L Boesby, N Juel Christensen, L Østergaard Kristensen

# *Medicinsk behandling af gastrointestinal blødning fra angiodysplasier*

# (kasuistik, planlægges indsendt primo november 2006 til Ugeskrift for læger)

#

# Kursusdeltagelse

# Prægraduat Kommunikationstræning i Laboratoriet for Kliniske Færdigheder, RH

# Postgraduat PhD kursus: GCP and Investigator-initiated Clinical Trials

# *Københavns Universitet, November 2006*

# Kursus vedr. lungeemboli, behandling og udredning

# *Dansk Cardiologisk Selskab, marts 2006*

#

# Læring i praksis (pædagogik II)

# *Center for Klinisk Uddannelse, RH, februar 2006*

#

# Ledelse, administration og samarbejde (LAS 1)

# *FoQUS, januar 2006*

#

# Kursus i patientkommunikation I og II

# *Dansk Institut for Medicinsk Simulation, KAS Herlev, april 2005*

#

# Kursus i akutkommunikation

# *Dansk Institut for Medicinsk Simulation, KAS Herlev, april 2005*

#

# Kursus i akut medicin

# *Dansk Institut for Medicinsk Simulation, KAS Herlev, oktober 2004*

#

# Læring i praksis (pædagogik)

# *Dansk Institut for Medicinsk Simulation, KAS Herlev, maj 2004*

#

# Læring i praksis (pædagogik)

# *Dansk Institut for Medicinsk Simulation, KAS Herlev, maj 2004*

#

# Kursus i akut kirurgi

# *Dansk Institut for Medicinsk Simulation, KAS Herlev, april 2004*

#

# Diverse Udarbejdelse af kittelinstruks til urologiske forvagter.

# KAS Herlev juni 2004

#

#

#

# Medlemskab af videnskabelige selskaber

#

# April 2006 ansøgt om medlemskab i Dansk Nefrologisk Selskab
